# CMOS‐Inspired Complementary Fluidic Circuits for Soft Robots

**DOI:** 10.1002/advs.202100924

**Published:** 2021-08-29

**Authors:** Sukho Song, Sagar Joshi, Jamie Paik

**Affiliations:** ^1^ Reconfigurable Robotics Laboratory École Polytechnique Fédérale de Lausanne (EPFL) Lausanne 1015 Switzerland

**Keywords:** analog fluidic circuits, complementary metal‐oxide‐semiconductor‐inspired fluidic circuits, controllable fluidic self‐oscillator, electronics‐free controllers, soft robotics

## Abstract

The latest efforts in digital fluidic circuits’ research aim at being electronics‐free, light‐weight, and compliant controllers for soft robots; however, challenges arise to adjust the fluidic circuit's digital logic operations. Currently there is no other way to modulate the amplitude or frequency but to structurally redesign the entire fluidic circuitry. This is mainly because there is currently no method to create an analog circuit‐like behavior in the digital fluidic circuits using conventional digitized fluidic gates. In this work, a new approach is presented to designing a circuit with digitized fluidic gates that is comparable to an analog circuit capable of actively tuning the circuit's fluidic characteristics, such as pressure gain, amplitude of output, and time response. For the first time, a pressure‐controlled oscillator is modeled, designed, and prototyped that not only controls the fluidic oscillation, but also modulates its frequency using only a single, quasi‐static pressure input. It can also demonstrate the circuit's performance for the control of a soft robotic system by actively modulating the motion of a soft earthworm robot up to twice of crawling speeds. This work has distinct contributions to designing and building intelligent pneumatic controllers toward truly comprehensive soft robotic systems.

## Graphical abstract

This paper reports a complementary metal‐oxide‐semiconductor‐inspired fluidic circuit design and components to actively control analog properties of digital fluidic logics. The proposed method enables to develop the first controllable fluidic self‐oscillation circuit that cannot only switch between “crawling” and “stop” status of a soft robot, but also can change the robot's crawling speed only using a single pressure input.

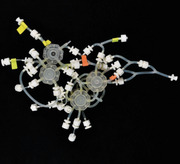

## 1 Introduction

Robotic systems combined with soft and functional components have shown high potentials to revolutionize how machines interact with human‐centered^[^ ^1^, ^2^, ^3^ ^]^ or unstructured environments.^[^ ^4^, ^5^, ^6^, ^7^ ^]^ For human scale applications, pneumatically‐driven soft actuators made of polymeric chambers have been one of the most favorable options to develop light, safe and interactive robotic systems designed for wearable prosthetic devices,^[^ ^8^, ^9^, ^10^ ^]^ or mobile soft robots.^[^ ^11^, ^12^, ^13^ ^]^ However, there remain practical and immediate challenges before application of the soft pneumatic actuators (SPAs) in soft robots toward everyday lives, as their driver circuits still consist of rigid electronics and bulky regulators, undermining their major advantage of mechanical compliance and customizability.

In line with the research efforts of developing soft robots whose control and actuation systems are entirely made out of soft materials analogous to biological systems in nature, microfluidic digital logics^[^ ^14^, ^15^ ^]^ have been of great interest^[^ ^16^, ^17^, ^18^ ^]^ in the field of soft robotics. Several previous works have shown high potential of the digital fluidic circuits as electronics‐free, lightweight, and compliant fluidic controllers.^[^ ^19^ ^]^ For example, Wehner et al. first demonstrated the design and manufacturing of an octopi‐shaped soft robot with an on‐board fluidic self‐oscillator to alternate actuation of eight legs.^[^ ^16^ ^]^ Also, Rothemund et al. developed a fully soft, bistable valve, and used it to build a fluidic self‐oscillator to generate the undulating motion of a soft crawling robot.^[^ ^20^ ^]^ Furthermore, Mahon et al. exploited the principle of digital fluidic logic circuits to build an on‐board fluidic controller to manipulate the gait motion of a hexapod robot.^[^ ^18^ ^]^

Despite of these promising results, the traditional digital fluidic logic circuits have failed to address three fundamental limitations to be used as a practical fluidic controller for soft robots. The first limitation is “lack of controllability”. Although the digital fluidic logics in previous works have successfully demonstrated their ability to enable soft robots to achieve highly intelligent maneuvers without relying on bulky electronics, there is currently no method to actively control the digital logic's analog properties, such as a speed of logic operations or a fluidic gain between input and output. This is the fundamental reason of what those previous fluidic self‐oscillation circuits mentioned above could do was nothing but generating a fluidic oscillation at a fixed oscillation frequency until the fluidic source is depleted.^[^ ^16^, ^20^ ^]^ The second limitation is “the need of excessive fluidic and electronic peripherals”. In order to achieve more controllability, the traditional fluidic logic circuits required multiple pressure sources and control inputs. For example, the fluidic logic controller used in the hexapod robot designed by Mahon et al. required “clock” and “input”, in addition to “vacuum source”, to create two “walking” and “grasp” status.^[^ ^18^ ^]^ Also, a fluidic digital‐to‐analog converter (DAC) demonstrated by Preston et al. required four different pneumatic supply pressures and two control inputs to digitally regulate an actuation pressure for a soft pneumatic gripper, which could have been done with one supply and one control inputs by a fluidic amplifier.^[^ ^21^ ^]^ In fact, Takao and Ishida demonstrated a complementary metal‐oxide‐semiconductor (CMOS)‐inspired pressure inverting amplifier.^[^ ^22^ ^]^ However, their amplifier still required both positive and negative pressure differential to create the CMOS‐inspired fluidic circuit. As shown in those previous works, the need of multiple pressure sources and inputs could effectively increase the number of rigid, bulky, and heavy, fluidic, and electronic peripherals in soft robots. The final limitation is “constant power dissipation”. As discussed by Preston et al., the traditional fluidic circuits based on Transistor‐Transistor‐Logic (TTL) that consist of only normally‐ON valves are destined to leak fluidic power at a steady state.^[^ ^21^ ^]^ Considering the fluidic power source will require a significantly larger amount of physical dimensions compared to electrical batteries, this constant power loss will pose a great limitation for future soft robotic applications.

As a possible solution to address the above challenges, in this paper, we propose a new fundamental approach to building “fluidic circuits” from fluid valves inspired by how conventional CMOS architecture is used for designing electric circuits. In electronics, CMOS circuits solve the constant power dissipation in the idle state, by pairing a p‐channel metal‐oxide‐semiconductor (PMOS) field‐effect transistor (FET) with an n‐channel metal‐oxide‐semiconductor (NMOS) FET that work complementary to each other. In the traditional microfluidics, a diaphragm valve known as “Quake valve” was used as a fluidic transistor; when exposed to a positive supply pressure differential, the valve behaves as normally‐ON fluidic gate, while a negative supply pressure differential makes the valve to be normally‐OFF fluidic gate.^[^ ^23^, ^24^, ^25^ ^]^ This means that a CMOS‐inspired fluidic circuit consisting of only Quake valves needs to incorporate electronic and fluidic peripherals associated with both positive and negative pressure differential. To the best of our knowledge, there does not exist a CMOS‐inspired fluidic circuit that consists of two different types of fluidic gates under a single pressure source. Therefore, we first define a normally‐OFF fluidic gate to be paired with the normally‐ON Quake valve under the same pressure source. The CMOS‐inspired fluidic circuits with these gates do not require additional peripherals for two different types of source and control pressure. Additionally, we discuss how to create analog circuit‐like behavior using fluidic gates with a non‐linear and digitized response. Unlike electronic transistors, traditional fluidic gates in microfluidics have shown a latching valve‐like behavior with fluidic hysteresis, allowing only “1” (full‐flow) or “0” (zero‐flow). Although the fluidic hysteresis has long been considered to be advantageous for digital fluidic logics due to its high noise resistance,^[^ ^26^ ^]^ such digitized fluidic response causes difficulties in building a fluidic analog circuit that is responsive to a continuous input. In this work, we propose a design methodology that works with such digitized fluidic gates for building fluidic analog circuits. Without having to reinvent new fluidic gates, our design method can combine the conventional digital fluidic logics with fluidic analog circuits to actively control the logic's gain, amplitude, or time response.

In the following sections, we first showed normally‐ON and normally‐OFF fluidic gates used in this work, as well as their fluidic characteristics. Instead of completely reinventing new fluidic structures, we exploited existing valve structures from conventional pneumatic components for the normally‐ON and normally‐OFF fluidic gates, which could be manufactured by commercialized additive manufacturing with a minimal effort of the manual assembly process. Using the normally‐ON and normally‐OFF fluidic structures, we built a CMOS‐inspired fluidic XOR logic to verify the given fluidic gates can be paired complementary to each other for a wide variety of CMOS‐inspired digital fluidic logic configurations. In the next step, we explained design principles of CMOS‐inspired complementary fluidic analog circuits that could actively change the circuit's pressure gain, amplitude, and hysteresis, working similar to a CMOS inverter‐based amplifier. By combining the CMOS‐inspired XOR fluidic logic with the CMOS‐inspired fluidic inverter amplifier, we built a pressure‐controlled oscillator (PCO) that worked similar to a voltage‐controlled oscillator (VCO) in electronics, demonstrating the potential of our approach to replicating a broad range of functionalities of various complex analog circuits in CMOS electronics. For example, unlike the traditional fluidic oscillators that need to be redesigned and remanufactured to change their frequency of fluidic oscillation,^[^ ^20^, ^27^, ^28^, ^29^ ^]^ the PCO developed in this work could actively control its oscillation frequency using a single control pressure input. To the best of our knowledge, our PCO circuit was the first controllable pneumatic self‐oscillator. Finally, we demonstrated the implementation of the PCO circuit for complex motion control of an earthworm‐inspired soft crawling robot. discussing how the outcomes in this work could bring potential impacts in achieving a high level of embedded physical intelligence for interactive soft robotic devices in the future.

## 2 Results

### 2.1 Experimental Characterization of Complementary Fluidic Gates

**Figure 1:**
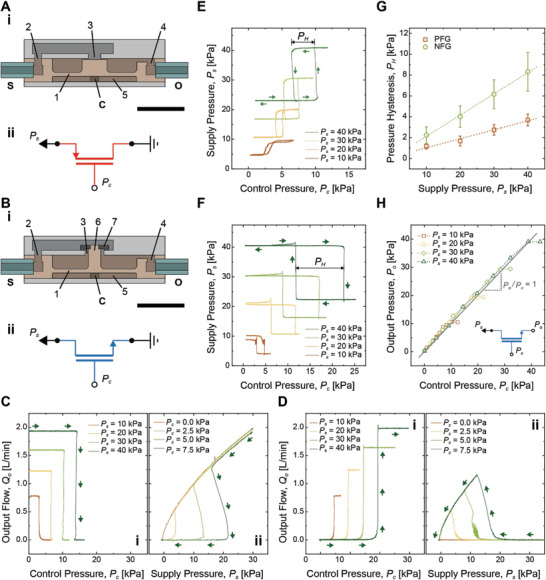
Cross‐sections of 3D assembly of the A‐i) PFG and the B‐i) NFG, and corresponding symbols in CMOS electronics (A‐ii and B‐ii, respectively). 1) diaphragm membrane, 2) inlet, 3) center opening, 4) outlet, 5) control chamber, 6) poppet shaft, 7) poppet. S: supply, O: output, C: control input. Detailed dimensions can be found in Figure S1, Supporting Information. Scale bars indicate 10 mm. C) Transfer (i) and output (ii) characteristics of the PFG. D) Transfer (i) and output (ii) characteristics of the NFG. Arrows indicate movements of output flow (*Q*_o_) profile with respect to *P*_c_ (i) and *P*_s_ (ii). *Q*_o_ is measured in (i) with *P*_c_ increases, while *P*_s_ decreases in (ii) following the arrow. E) Profiles of supply pressure (*P*_s_) of the PFG, depending on control pressure (*P*_c_). F) Profiles of *P*_s_ of the NFG, depending on *P*_c_. Arrows indicate movements of *P*_s_ followed by the change in *P*_c_. G) Pressure hysteresis (*P*_H_) on the PFG and the NFG as a function of *P*_s_. Each point indicates an average of four measurements (n = 4) with corresponding error bars of ±1 SD. H) Output pressure (*P*_o_) of the NFG, depending on *P*_s_ and *P*_c_, with the output closed. A grey line indicates a gain of unity (*P*_o_/*P*_c_ = 1).

In this work, we exploit existing fluidic valve structures to define normally‐ON and normally‐OFF fluidic gates that are complementary to each other in logic circuits as shown in **Figure** **1**. To have close correlations to the PMOS and the NMOS transistors, we call a fluidic equivalent to the PMOS transistor a p‐channel fluidic gate (PFG), while an equivalent to the NMOS transistor an n‐channel fluidic gate (NFG). Unlike electronics, fluidic flow does not have p‐type or n‐type polarities. Therefore, we define the PFG and the NFG based on the gate's behavior in logic operation, as suggested in previous works. For example, a fluidic gate is defined as a PFG if the gate is normally‐ON status and closes with a control input.^[^ ^28^ ^]^ Similarly, a fluidic gate is defined as an NFG if the gate is normally‐OFF status and opens with a control input.^[^ ^27^ ^]^ The PFGs and the NFGs are manufactured by conventional multi‐material 3D printing with a combination of rigid and rubber‐like materials, connected through soft tubing (Figure 1A,1B). Specific dimensions of the PFG and the NFG are shown in Figure S1, Supporting Information. For the PFG, we use a Quake‐style fluidic gate (Figure 1A‐i). The diaphragm check valve introduced by Unger et al.^[^ ^14^ ^]^ is the most popular microvalve in the field of microfluidics due to its simple structure and high compatibility to a broad range of manufacturing from 3D printing,^[^ ^30^, ^31^ ^]^ microfabrication,^[^ ^32^, ^33^ ^]^ to soft lithography.^[^ ^34^ ^]^ The Quake‐style fluidic gate has already been used as a PFG in previous works, since it functions as a normally‐ON switch.^[^ ^28^ ^]^ The PFG consists of inlet (2) and outlet (4), as well as an elastomeric diaphragm membrane (1) that closes center opening (3) to block an airflow from the supply (S) to outlet (O) by a positive control pressure (*P*_c_) in the control chamber (5).

For the NFG, on the other hand, we use an existing fluidic structure of a poppet valve (Figure 1B‐i). Connected to a poppet (7) with a poppet shaft (6), the membrane in the NFG opens center opening (3), whose effective size is equivalent to the center opening of the PFG. Although the fluidic structure of the poppet valve prevails in conventional pneumatic components, there doesn't exist a previous work in microfluidics that exploits the poppet valve as the NFG to configure CMOS fluidic logic. In fact, Perdigones et al. have developed a microvalve that shows NMOS‐like behavior that enhances the output flow with respect to increased control pressure.^[^ ^35^ ^]^ However, this valve does not function as a normally‐OFF valve to completely block a supply pressure, thus there always exists fluidic leakage. This makes the Perdigones's valve incompatible to be paired with the normally‐ON Quake‐style microvalve. On contrary, the poppet‐style NFG in this work shows fluidic characteristics as a normally‐OFF valve, which only creates a fluidic channel from the supply to the output when control input reaches a threshold.

Fluidic transfer and output characteristics of the PFG and NFG in Figure 1C,1D show both similarities and differences between the fluidic gates and electronic transistors. Here, transfer characteristics show output flow (*Q*_o_) with respect to control pressure (*P*_c_), while output characteristics present output flow (*Q*_o_) with respect to supply pressure (*P*_s_). As shown in Figure 1C, the PFG possesses fluidic characteristics analogous to the p‐channel depletion mode MOSFET in electronics with opposite polarities in *Q*_o_ and *P*_c_, as there is no carrier polarity in fluidics. In case of the PFG, *Q*_o_ shows positive under positive *P*_s_, and its threshold *P*_c_ is also positive. Figure 1C‐i shows the PFG stays opened at *P*_c_ = 0 kPa regardless of *P*_s_ and closes at a positive *P*_c_. Figure 1C‐ii shows a range of *P*_s_ staying closed (*Q*_o_ = 0) expands with an increase in *P*_c_, similar to the PMOS reducing the source‐drain current (*I*_DS_) with respect to the increased negative gate voltage (*V*_GS_). On the other hand, the NFG possesses fluidic characteristics analogous to the n‐channel enhancement‐mode MOSFET (Figure 1D). The NFG opens at a positive threshold *P*_c_, allowing positive *Q*_o_ under positive *P*_s_ (Figure 1D‐i). The range of *P*_s_ having the NFG opened also expands with the increase in *P*_c_ (Figure 1D‐ii), similar to the NMOS enhancing *I*_DS_ with respect to *V*_GS_.

The biggest difference in transfer and output characteristics between the fluidic gates and the electronic transistors comes from the digitized fluidic response of the fluidic gates. *Q*_o_ in Figure 1C‐i,1D‐i shows step responses to *P*_c_, while *I*_DS_ characteristics of the PMOS and the NMOS in electronics show continuous changes with respect to *V*_GS_. This means that *Q*_o_ in the saturation region does not vary with respect to the applied *P*_c_ (Figure 1C‐ii,1D‐ii). The PFG shows the same *Q*_o_ profiles with respect to *P*_s_ for all *P*_c_ once the gate opens. The NFG completely closes with *Q*_o_ = 0 at a high *P*_s_ regardless of *P*_c_. Therefore, those digitized fluidic gates cannot continuously regulate *Q*_o_ by means of *P*_c_.

Instead, we found that fluidic hysteresis of the gates can be continuously regulated by *P*_s_. Figure 1E,1F shows hysteric behavior of *P*_s_ in the PFG and the NFG depending on *P*_c_, which is a common characteristic of the fluidic gates reported in previous works.^[^ ^20^, ^21^, ^23^, ^24^, ^26^, ^27^, ^29^, ^36^ ^]^ In case of the PFG with its output opened (Figure 1A‐ii), *P*_s_ begins with 23.0 kPa at *P*_c_ = 0 kPa, as the air escapes to the ambient air through the output. When *P*_c_ reaches to 10.2 kPa, the membrane moves upward to close the center opening, resulting in an acute increase in *P*_s_ up to *P*_s_ = 40 kPa. Once the gate is closed, the area difference between the center opening and the membrane allows a small *P*_c_ to keep the PFG closed. The PFG reopens at *P*_c_ = 6.6 kPa, resulting in a pressure hysteresis *P*_H_ = 3.6 kPa. In case of the NFG with its output opened (Figure 1B‐ii), on the other hand, the NFG opens the gate at *P*_c_ = 22.5 kPa, leading to *P*_s_ dropping down from 40 to 22.3 kPa. The reduced *P*_s_ allows the NFG to remain open until *P*_c_ decreases to *P*_c_ = 11.8 kPa, resulting in *P*_H_ = 10.8 kPa (Please follow the arrows in Figure 1E,1F). Figure 1G shows that the amount of *P*_H_ on both PFG and NFG is proportional to the applied *P*_s_, while the NFG shows greater *P*_H_, compared to that of the PFG.

A key advantage of using the proposed poppet structure as the NFG is that we can use it as a pressure regulator to actively change the *P*_s_. In Figure 1H, we blocked the output port of the NFG and measured its saturated, quasi‐static output pressure (*P*_o_), depending on *P*_c_. As seen in Figure 1H, *P*_o_ increases in proportional to the applied *P*_c_ with a gain of unity (*P*_o_ / *P*_c_ = 1), until it reaches to the supplied *P*_s_. When *P*_c_ is applied, the NFG opens the valve until *P*_o_ reaches to *P*_c_, as shown in Figure S2A, Supporting Information. In the case of the PFG, on the other hand, *P*_o_ always reaches to *P*_s_ regardless of *P*_c_, as shown in Figure S2B, Supporting Information. Therefore, using the NFG as a pressure regulator, we can locally control *P*_s_ of a specific fluidic gate to change its output pressure and fluidic hysteresis. In electronics, an NMOS transistor that regulates the output voltage with a gain of unity is called a voltage follower. Therefore, Figure 1H shows that the poppet‐style valve can indeed function the same as the NMOS transistor in electronics, which is a perfect complementary fluidic gate to be paired with the PFG. In this work, we call an NFG a “pressure follower”, when specifically used for regulating the output pressure, analogous to the voltage follower in electronics.

**Figure 2:**
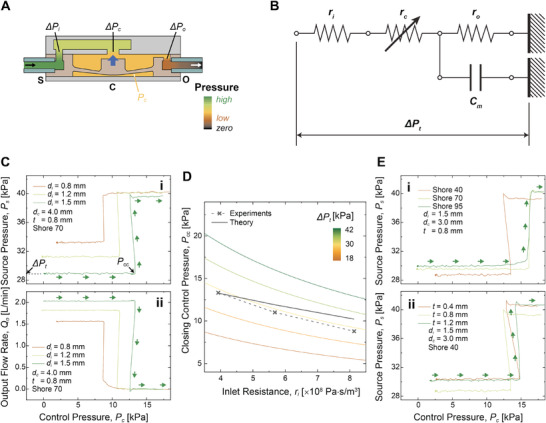
Fluidic characteristics of the PFG with various design parameters. A) Schematic images of pressure distribution inside the fluidic channel of an opened PFG. B) A lumped‐element model of electronic components equivalent to the opened PFG. C) Profiles of i) *P*_s_ and ii) *Q*_o_ of the PFG with respect to *P*_c_, depending on various inlet diameters (*d*_i_). D) Closing control pressure (*P*_cc_) with respect to inlet resistance (*r*_i_), depending on various total pressure drops (∆*P*_t_). E) Profiles of *P*_s_ of the PFG with respect to *P*_c_, depending on i) membrane's shore stiffness and ii) thickness of the membrane (*t*). Arrows indicate movements of *P*_s_ profile followed by the change in *P*_c_.

As reported in previous works, an opened fluidic gate can be expressed by a lumped‐element model of electronic components^[^ ^37^, ^38^ ^]^ (**Figure** **2**A). In this work, based on the lumped model, we assume the flow to be incompressible, quasi‐static (d*Q*_o_ */*d*t* = 0), and perfect laminar flow from the supply to the output. Also, we only consider fluid resistance of inlet hole (*r*_i_), center opening (*r*_c_), and outlet hole (*r*_o_) as shown in Figure 2B. Note that *r*_c_ is given as a variable resistance, since its fluid resistance changes with respect to the movement of the diaphragm membrane. A fluid resistance (*r*) of a circular hole can be described with Hagen–Poiseuille law,^[^ ^37^ ^]^

(1) $$r=128\eta l/\pi d^{4}$$

As shown in Equation (1), the fluid resistance of a hole is proportional to the thickness of the hole (*l*) and viscosity of the fluid (*η*), while inversely proportional to the hole diameter (*d*) to the power of four. Meanwhile, the diaphragm membrane can store hydraulic energy by means of elastic deformation similar to a capacitor in electronics, whose capacitance (*C_m_*) can be described as,

(2) $$C_{m}=\pi a^{6}\left(1-\nu^{2}\right)/16E_{m}t^{3}$$

Here, *a*, *E_m_*, *t*, and *ν* are radius, Young's modulus, thickness, and Poisson's ratio of the membrane, respectively.^[^ ^38^ ^]^ As shown in Figure 2B, the total pressure drop (Δ*P_t_*) across the opened PFG can be expressed as a sum of pressure drop caused by the fluid resistance from the inlet, center opening, and outlet.

(3) $$\Delta P_{t}=\left(r_{i}+r_{c}+r_{o}\right)\;Q_{o}$$

A closing control pressure (*P*_cc_) defined as an air pressure to close the gate will be equal to the pressure drop across the outlet (Δ*P*_o_) acting on top of the diaphragm membrane, which is given as,

(4) $$P_{\mathrm{cc}}=\Delta P_{o}\;=\Delta P_{t}\cdot r_{o}/\left(r_{i}+r_{c}+r_{o}\right)$$

Figure 2C shows the effect of inlet diameter (*d*_i_) on the profile of *P*_s_ and *Q*_o_ with respect to *P*_c_. As seen in Figure 2C‐i, Δ*P_t_* is equal to *P*_s_ of the opened fluidic gate (*P*_c_ = 0). Δ*P*_t_ increases from 29 to 33 kPa with respect to a decrease in *d*_i_, as it results in a higher *r_i_* that increases the sum of fluid resistance (*r_i_* + *r_c_* + *r_o_*) in Equation (3). Meanwhile, the decrease in *d*_i_ reduces *P*_cc_ from 13 to 9 kPa, as the denominator in Equation (4) increases with a higher *r_i_*.

The Hagen–Poiseuille law^[^ ^37^ ^]^ shown in Equation (1) is valid for an incompressible fluid running through a simple circular tubing, but is not suitable for compressible gas flow inside the PFG with an intricate fluidic channel. Instead, we estimate *r_i_*, *r_c_*, and *r_o_* from the experimental observations in Figure 2C using Equations (3) and (4), as shown in Table S1, Supporting Information. Using the estimated *r_i_*, *r_c_*, and *r_o_*, we predict *P*_cc_ for various Δ*P*_t_ with *r_i_*, as shown in Figure 2D. The calculated *P*_cc_ (black solid line) well matches with the experimental results (dashed line with cross) with less than 1.5 kPa margin. Also, the calculation shows decreased *P*_cc_ when *r_i_* increases (smaller *d*_i_), which matches with the experiments shown in Figure 2C. The above results verify that the proposed equations can explain fluidic mechanics inside the fluidic gate, and the simple lumped model will provide good theoretical estimations on characteristics of the fluidic gates, as long as we can estimate precise fluid resistance using experiments or FEM analysis.

Note that the capacitive component which is a function of mechanical properties of the membrane does not play a role in *P*_cc_ as shown in Equation (4). Experimental results shown in Figure 2E also confirm that there is no noticeable change in the actual *P*_s_ profiles with respect to stiffness or thickness of the diaphragm membrane. This result also implies we can further reduce the size of the fluidic gate without changing its fluidic characteristics, which will allow us to miniaturize the overall size of fluidic circuits as a future work.

### 2.2 Experimental Verification of CMOS‐Inspired Fluidic Digital Logics

**Figure 3:**
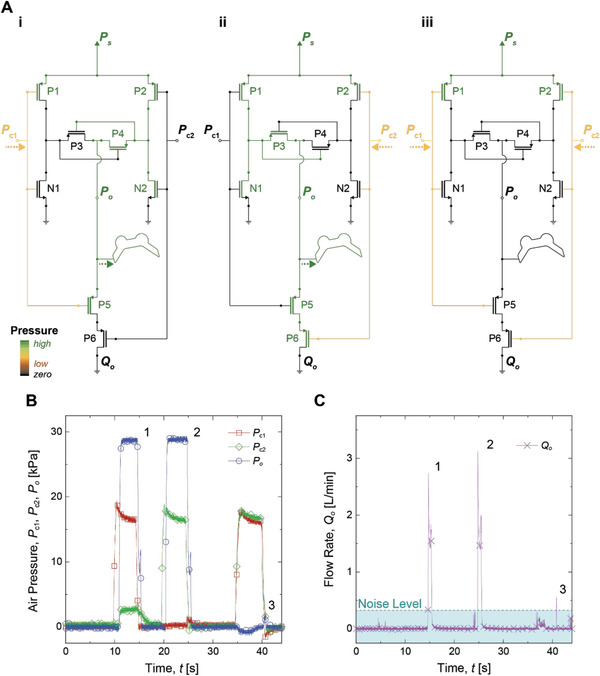
CMOS‐inspired pneumatic XOR logic consisting of PFGs and NFGs A) schematic pressure maps of XOR logic diagram, in case of i) activation of *P*_c1_, ii) activation of *P*_c2_, and iii) activation of both *P*_c1_ and *P*_c2_. Dashed lines display pressurization of two control input (*P*_c1_ and *P*_c2_) and output pressure (*P*_o_). B) *P*_o_ of the pneumatic XOR, depending on *P*_c1_ and *P*_c2_. C) Flow rate at the output of NOR logic P6 (*Q*_o_), depending on *P*_c1_ and *P*_c2_. 1) activation of *P*_c1_, 2) activation of *P*_c2_, 3) activation of both *P*_c1_ and *P*_c2_.

The normally‐OFF NFG that works complementary to the conventional Quake‐style gates (PFG) allows translating various CMOS‐based digital logics in electronics to fluidic domain. **Figure** **3** demonstrates an example of fluidic digital logics composed of the PFGs and the NFGs. Figure 3A shows schematic pressure maps of a pneumatic XOR logic depending on different control inputs *P*_c1_ and *P*_c2_. The CMOS XOR logic consists of several sub‐logics; two pneumatic inverters P1N1 and P2N2, PFGs P3 and P4 working complementary to each other, and a NOR logic P5P6. When *P*_c1_ is activated (Figure 3A‐i and 1 in Figure 3B), P1N1 depressurizes P3, while P4 remains pressurized by P2N2. The air from P4 pressurizes a SPA with supply pressure *P*_s_. Finally, *P*_c1_ blocks P5 in the NOR logic to prevent output pressure (*P*_o_) from leaking. When *P*_c2_ is activated (Figure 3A‐ii and 2 in Figure 3B), on the other hand, P2N2 depressurizes P4, while P3 remains pressurized by P1N1, and *P*_c2_ closes P6 to prevent *P*_o_ from leaking. In case both *P*_c1_ and *P*_c2_ exist (Figure 3A‐iii and 3 in Figure 3B), two NOT logics P1N1 and P2N2 depressurize both P3 and P4, resulting in complete depressurization of the XOR logic. Figure 3B,3C shows profiles of *P*_c1_, *P*_c2_, and *P*_o_, as well as flow rate at the output of NOR logic P6 (*Q*_o_) with respect to time (*t*). As shown in Figure 3B, the XOR logic produces *P*_o_, when either *P*_c1_ or *P*_c2_ is pressurized. Note that only 19 kPa of *P*_c1_ or *P*_c2_ can regulate *P*_o_ of 29 kPa. A pressure gain (*P*_o_|_max_/*P*_c_|_max_) defined as a ratio of the maximum *P*_o_ (*P*_o_|_max_) divided by the maximum *P*_c_ (*P*_c_|_max_) is approximately 1.5, greater than unity (*P*_o_|_max_/*P*_c_|_max_ > 1).

In case both *P*_c1_ and *P*_c2_ are depressurized from 19 kPa (3 in Figure 3B), the XOR logic shows a negligible leakage of *P*_o_ = 2 kPa, which can also be seen in Figure 3C. Since the PFG and the NFG show a step response to a control input as shown in Figure 1C,1D, we speculate that a small difference in fluidic characteristics among fluidic gates can result in such a transition leakage. Figure 3C and Movie S2, Supporting Information, show that the CMOS‐inspired fluidic XOR logic composed of several sub‐logics consumes the pressurized air, only when the logic status changes, providing proof that any CMOS logics in electronics can be replicated by a combination of the PFGs and the NFGs.

### 2.3 Active Tuning of Analog Properties of CMOS‐Inspired Fluidic Logics

**Figure 4:**
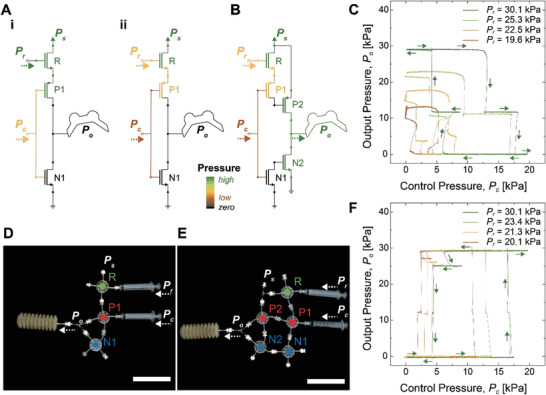
Active hysteresis and gain control of CMOS‐inspired pneumatic inverter amplifiers. A) Schematic pressure maps of a pneumatic inverter P1N1 with a pressure follower R, i) in case of a high regulator pressure (*P*_r_) (i.e., *P*_r_ = 30.1 kPa in 3C) and ii) a low *P*_r_ (i.e., *P*_r_ = 19.6 kPa in 3C). Note that the SPA is depressurized with a control pressure (*P*_c_), as the P1N1 works as a NOT logic. B) A schematic pressure map of a cascaded inverter circuit (R‐P1P2N1N2) at an optimal *P*_r_ (i.e., *P*_r_ = 20.1 kPa in 3F). A high supply pressure (*P*_s_) of 29.3 kPa can be controlled with a much lower *P*_c_ = 10.9 kPa, compared to *P*_c_ = 17.0 kPa in a single‐stage inverter P1N1 (see Figure 3A‐i). C) Profiles of *P*_o_ of the single‐stage inverter amplifier (R‐P1N1) with respect to *P*_c_, depending on *P*_r_. D) A photo image of the single‐stage pneumatic inverter R‐P1N1. E) A photo image of a cascaded pneumatic inverter (R‐P1P2N1N2). F) Profiles of *P*_o_ of the cascaded pneumatic inverter (R‐P1P2N1N2) with respect to *P*_c_ depending on *P*_r_. The scale bars indicate 10 cm, and dashed lines display activation of *P*_c_, *P*_r_, and *P*_o_.

In CMOS‐inspired fluidic circuit configurations, the use of an NFG as a pressure regulator can provide a key element in replicating analog circuit‐like behaviors with fluidic gates that show non‐linear, digitized fluidic responses. **Figure** **4**A,4D shows a schematic pressure map and a photo of a pneumatic inverter P1N1 with a pressure follower R. The pressure follower R takes a separate control input, which is defined as a regulator pressure (*P*_r_). 29.1 kPa of a supply pressure (*P*_s_) is fed to the pressure follower R, whose output pressure becomes the supply pressure of the pneumatic inverter P1N1. Figure 4C shows profiles of output pressure (*P*_o_) of the R‐P1N1 circuit with respect to a control pressure (*P*_c_), depending on *P*_r_ ranging from 19.6 to 30.1 kPa. In the case of *P*_r_ = 30.1 kPa, for example, the SPA is initially pressurized with *P*_o_ = 29.1 kPa at *P*_c_ = 0 kPa, as the inverter P1N1 works as a NOT gate in CMOS logic. *P*_o_ of the P1N1 becomes zero at *P*_c_ = 17.9 kPa (Figure 4A‐i) and returns to 29.1 kPa at *P*_c_ = 4.2 kPa. When *P*_r_ decreases (Figure 4A‐ii), the reduced *P*_r_ allows a less *P*_o_ that can be depressurized with a smaller *P*_c_ (see *P*_r_ = 22.5 kPa in Figure 4C, for example). It is important to note that the smaller *P*_o_ also results in a reduced pressure hysteresis (*P*_H_). As shown in Figure 4C, *P*_H_ at *P*_r_ = 30.1 kPa decreases from 13.7 to 5.2 kPa, 61.7% of reduction when *P*_r_ = 19.6 kPa. *P*_r_ also changes pressure gain. A pressure gain (*P*_o_|_max_/*P*_c_|_max_) of the P1N1 increases up to 2.4 at *P*_r_ = 19.6 kPa, compared to 1.6 at *P*_r_ = 30.1 kPa. The pressure gains greater than unity (*P*_o_|_max_/*P*_c_|_max_ > 1) indicate that the pneumatic circuit P1N1 amplifies a low *P*_c_ to control a high *P*_s_, working analogously to a CMOS inverter‐based amplifier.

Using a cascaded logic architecture shown in Figure 4E, we can further multiply the pressure gains of each pneumatic inverters, analogous to Darlington‐pair transistors in electronics.^[^ ^39^ ^]^ A schematic pressure map Figure 4B and Movie S3, Supporting Information, show the concept of gain multiplication. In Figure 4B, two pneumatic inverters P1N1 and P2N2 are supplied with *P*_s_ = 29.3 kPa. An output of the first stage inverter P1N1 is connected to the second stage inverter P2N2 as a control input. Since an output of a NOT logic P1N1 becomes a control input of another NOT logic P2N2, the cascaded inverter in Figure 4B works as a normally‐OFF gate. Using the pressure follower R, we can reduce the output of the P1N1 just high enough to close the P2N2. The reduced output of the P1N1 allows the cascaded inverter to be controlled with a smaller *P*_c_ at a higher pressure gain, as discussed in Figure 4A‐ii. With an optimal *P*_r_ = 20.1 kPa, the cascaded pneumatic inverter can open and close *P*_s_ = 29.3 kPa at *P*_c_ of only 10.9 kPa (Figure 4F). This *P*_c_ is 64% smaller than the *P*_c_ of a single‐stage pneumatic inverter (≈17.0 kPa) running at the same *P*_s_. The maximum pressure gain (*P*_o_|_max_/*P*_c_|_max_) of the cascaded inverter is estimated to be 2.7, which is close to the multiplied pressure gain of two single inverter circuits, 1.6^2^ = 2.56.

### 2.4 Logic Architecture and Fluidic Characterization of Pressure‐Controlled Oscillator

**Figure 5:**
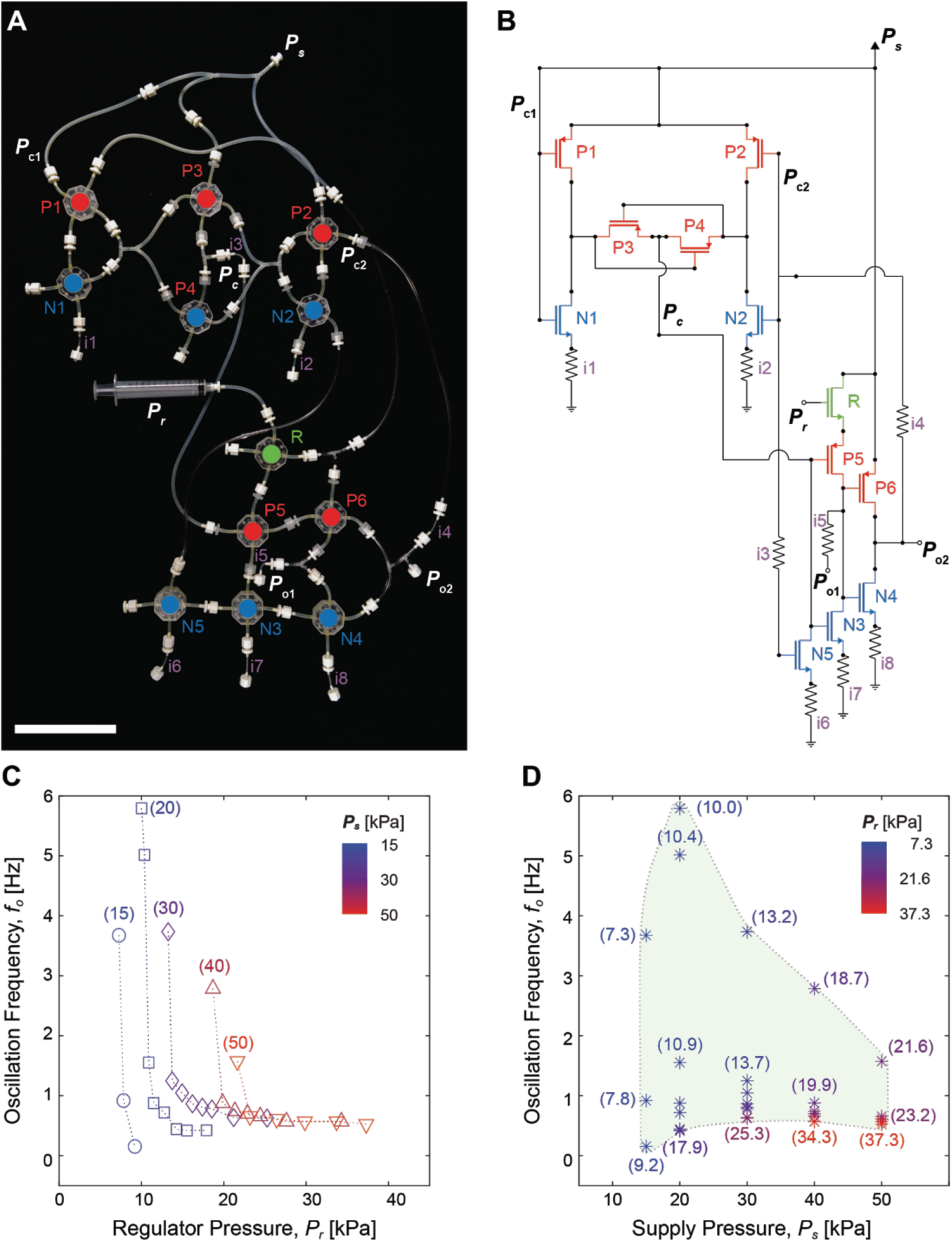
A PCO to replicate functions of VCO in CMOS electronics. A) A photo image of the circuit configuration of the PCO, B) and its circuit diagram. C) Oscillation frequencies (*f*_o_) of the PCO depending on the regulator pressure (*P*_r_) and D) the supply pressure (*P*_s_). Numbers in C and D correspond to *P*_s_ and *P*_r_, respectively.

Utilizing an NFG as a pressure follower in a CMOS‐inspired complementary circuit configuration composed of the proposed PFGs and NFGs, we can replicate functionalities of a complex analog circuit in fluidic domain. **Figure** **5**A shows a PCO that replicates a CMOS‐based VCO, which has not been accomplished in previous works. As shown in a circuit diagram Figure 5B, the PCO consists of the CMOS‐inspired fluidic XOR logic (P1P2P3P4N1N2), the CMOS‐inspired cascaded fluidic inverter (P5P6N3N4) with the pressure follower R, and the NFG N5 that functions as an exhaust regulator similar to the NOR gate P5P6 in Figure 3A. To achieve stable oscillation characteristics, we added fluidic resistors ≈i1–i8 made out of a silicone tube with 0.8 mm in diameter and 20 mm in length into the PCO circuit to match fluidic impedances between the XOR logic and the cascaded inverter. The XOR logic receives the first control input (*P*_c1_) directly from a supply pressure (*P*_s_), while the second input (*P*_c2_) is connected to the output (*P*_o2_) of the second stage pneumatic inverter P6N4. In the case of regulator pressure *P*_r_ = 0 kPa, the output of the inverter P5N3 (*P*_o1_) is 0 kPa. The cascaded inverter works as a normally‐ON valve, resulting in *P*_c2_ = *P*_s_. Since *P*_c1_ and *P*_c2_ are both pressurized with the same *P*_s_, the output of the XOR (*P*_c_) becomes 0 kPa, preventing fluidic oscillation. When applied a *P*_r_, on the other hand, the pressure follower R allows the inverter P5N3 to produce an output *P*_o1_, consecutively closing the upper inverter P6N4, resulting in *P*_c2_ = 0 kPa. Since *P*_c1_ is always *P*_s_, a pressure difference between *P*_c1_ and *P*_c2_ allows the XOR logic to produce a *P*_c_. The increase in *P*_c_ eventually closes the first stage inverter P5N3 again, resulting in *P*_o1_ = 0 kPa and *P*_c2_ = *P*_s_. The pressure difference between *P*_c1_ and *P*_c2_ disappears, resulting in *P*_c_ = 0 kPa. This series of chain reactions generates a pneumatic self‐oscillation controlled by a single pneumatic input *P*_r_. As discussed in Figures 1G, 4C, and 4F, a decrease in *P*_r_ reduces a pressure hysteresis (*P*_H_) of the cascaded pneumatic inverter. The reduced *P*_H_ also reduces the time response between pressurization (ON‐status) and depressurization (OFF‐status) of the cascaded inverter, resulting in a faster oscillation compared to that with a higher *P*_r_.

Figure 5C,D and Table S2, Supporting Information, show oscillation frequencies (*f*_o_) of the PCO circuit in the unloading condition depending on *P*_r_ and *P*_s_, respectively. Figure 5C shows that *f*_o_ decreases with respect to an increase in *P*_r_, which is analogous to a CMOS‐based VCO in electronics.^[^ ^40^ ^]^ In case of *P*_s_ = 30 kPa, for example, the oscillation begins at *P*_r_ = 25.3 kPa with *f*_o_ = 0.6 Hz, and it becomes twice faster up to *f*_o_ = 1.2 Hz at *P*_r_ = 13.7 kPa. At *P*_r_ = 13.2 kPa, the slight decrease of 0.5 kPa in *P*_r_ produces 3 times faster oscillation up to *f*_o_ = 3.7 Hz. As seen in Figure 5D, *f*_o_ also varies with respect to *P*_s_, ranging from 15 to 50 kPa. The pneumatic circuit could not oscillate at *P*_s_ lower than 15 kPa. Also, the PCO shows a significant decrease in *f*_o_ at *P*_s_ higher than 50 kPa. The widest frequency modulation is achieved at *P*_s_ = 20 kPa, ranging from *f*_o_ = 0.4 Hz at *P*_r_ = 17.9 kPa to *f*_o_ = 5.8 Hz at *P*_r_ = 10.0 kPa.

**Figure 6:**
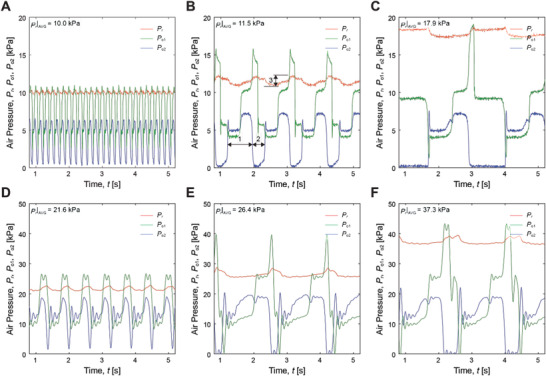
Fluidic characteristics of the PCO Profiles of regulator pressure (*P*_r_), as well as output pressures at the first stage P5N3 and the second stage pneumatic inverters P6N4 (*P*_o1_ and *P*_o2_, respectively), with respect to time (*t*). In case of a supply pressure *P*_s_ = 20 kPa, *P*_r_ varies from A) *P*_r_ = 10.0 kPa, B) *P*_r_ = 11.5 kPa, to C) *P*_r_ = 17.9 kPa. In case of *P*_s_ = 50 kPa, *P*_r_ varies from D) *P*_r_ = 21.6 kPa, E) *P*_r_ = 26.4 kPa, to F) *P*_r_ = 37.3 kPa.

Several distinctive features of the PCO are shown in **Figure** **6** with profiles of regulator pressure (*P*_r_), output pressure at the first stage inverter P5N3 (*P*_o1_), and output pressure at the second stage inverter P6N4 (*P*_o2_) with respect to time (*t*). Please note that the pneumatic oscillation causes fluctuation of *P*_r_ as shown at 3 in Figure 6B, despite the pressure follower R keeps pressurized with a constant syringe volume. Here we define *P*_r_|_AVG_ to be an average of *P*_r_.

Overall, Figure 6 shows that the peak of *P*_o1_ and *P*_o2_ appear with time delays, which can actuate multiple actuators with a shifted phase to generate peristaltic motion. For example, the phase difference between *P*_o1_ and *P*_o2_ varies in a range from 35° to 72° at supply pressure *P*_s_ = 20 kPa, and from 38° to 99° at *P*_s_ = 50 kPa, depending on *P*_r_. Duty cycle of the PCO can also vary with *P*_r_; as shown in Figure 6B for an example, a decrease in *P*_r_|_AVG_ reduces the depressurizing phase (2 in Figure 6B) greater than the pressurization phase (1 in Figure 6B), resulting in an increased duty cycle. In case of *P*_s_ = 20 kPa, the duty cycle increases from 52% to 70.3%, while it does from 75.1% to 87.7% at *P*_s_ = 50 kPa. Furthermore, the PCO can support two different modes of output pressure; one is a constant amplitude, and the other is a variable amplitude in output pressure. The maximum amplitude of *P*_o2_ (*P*_o2_|_max_) shows constant regardless of *P*_r_|_AVG_, while *P*_o1_|_max_ varies with respect to *P*_r_|_AVG_. In case of *P*_s_ = 20 kPa, for example, *P*_o1_|_max_ varies 8.1 kPa with respect to *P*_r_|_AVG_, while *P*_o2_|_max_ does only 1.1 kPa. In case of *P*_s_ = 50 kPa, *P*_o1_|_max_ changes 16.5 kPa with respect to *P*_r_|_AVG_, while *P*_o2_|_max_ does 3.3 kPa.

### 2.5 Active Motion Control of a Soft Robot Using the Pressure‐Controlled Oscillator

**Figure 7:**
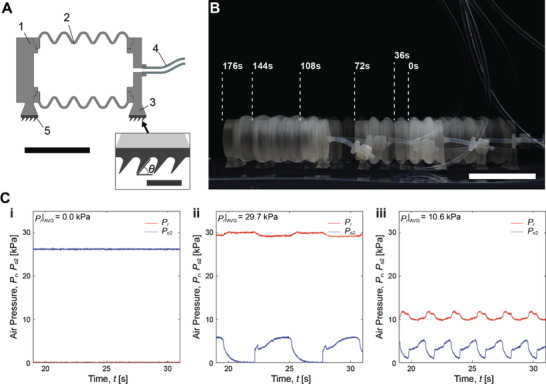
Design, control and, characterization of a pressure‐controlled soft robot. A) A 3D cross‐sectional image of an earthworm‐inspired soft crawling robot with a directional friction footpad and single DoF actuated body. 1) front‐cap, 2) bellow‐skin, 3) rear‐cap, 4) tubing, 5) gecko‐inspired directional wedge hair adhesives. The angle of the wedge hairs against flat surface (*θ*) is 70°. The scale bar outside the inset indicates 30 mm, while the bar inside the inset does 4 mm. B) A time‐lapse image of the earthworm‐inspired soft crawling robot. Dashed lines indicate positions of front‐end of the soft robot at every 36s crawling toward the left side of the page. The scale bar corresponds to 50 mm. C) Profiles of output pressure (*P*_o2_) and regulator pressure (*P*_r_) with respect to time (*t*), in case of i) *P*_r_|_AVG_ = 0, ii) 29.7, and iii) 10.6 kPa, which are the same oscillating conditions to 176, 36, and 108 s in (B).

As illustrated in **Figure** **7**, Movie S1 and Movie S4, Supporting Information, we implemented the PCO circuit to demonstrate a CMOS‐like complex fluidic analog circuit to control the crawling motion of a 3D printed earthworm‐like soft robot (Figure 7A) with gecko‐inspired directional adhesives (5 in Figure 7A) using a single input. In order to move this robot, it requires an alternating gait pattern associated with its body shape. Therefore, the soft robot changes its bellow‐shaped body length (2 in Figure 7A) upon pressurization. When pressurized, the forefoot moves forward, as an increase in body length pushes the directional adhesives on the forefoot against the tilting angle (*θ*). On contrary, the gecko adhesives on the aft foot bend along to the tilting angle (*θ*), resulting in an enhanced friction with increased contact area to keep the aft foot beholding the same position. When the bellow‐shaped body is depressurized, the directional adhesives behave in the opposite way; while the forefoot grips the surface due to the enhanced friction, the aft foot comes forward, as its foot hairs lose contact with the surface.

Figure 7C shows the profiles of regulator pressure (*P*_r_) and output pressure (*P*_o2_) of the PCO during locomotion. While pressurized with 26.2 kPa of a supply pressure (*P*_s_) during stand‐by, the soft robot does not consume any pressurized air due to the PCO's CMOS‐inspired complementary construction (176s in Figure 7B,7C‐i). Also, the PCO can switch between “crawling” and “stop” status using the regulator pressure without additional control inputs. The soft robot starts crawling with a low oscillation frequency (*f*_o_) up to *f*_o_ = 0.15 Hz at 29.7 kPa of a high regulator pressure in average (*P*_r_|_AVG_) (36s in Figure 6B,6C‐ii), and increases its crawling speed with a faster oscillation up to *f*_o_ = 0.40 Hz at *P*_r_|_AVG_ = 10.6 kPa (108s in Figure 7B,7C‐iii). Estimated crawling speed is initially 0.5 mm s^−1^ at *P*_r_|_AVG_ = 29.7 kPa and increases up to 1.0 mm s^−1^ at *P*_r_|_AVG_ = 10.6 kPa. Beyond the oscillation frequency faster than *f*_o_ = 0.40 Hz, the crawling speed does not increase, since deformation of the soft robotic body cannot keep up with a faster oscillation, resulting in the reduced stroke for the crawling motion.

## 3 Discussions and Conclusion

This work discusses the following design principles of CMOS‐inspired complementary fluidic analog circuits with conventional digitized fluidic gates, and its applications to empowering soft, interactive robotic systems. First, the existing fluidic structure of a poppet‐style, normally‐OFF valve is suitable to be paired with the Quake‐style, normally‐ON valve for CMOS‐inspired complementary fluidic logic and analog circuits. As shown in Figure 1, the poppet‐style, NFG perfectly functions as a complementary valve to the Quake‐style, PFG, in addition to its ability to regulate supply pressure, analogous to a voltage follower in electronics. We successfully replicate a CMOS‐inspired XOR logic using the PFG and the NFG in Figure 3, validating no power consumption in steady state. Also, the amount of gain and hysteresis of those fluidic gates can be controlled by regulating a supply pressure using the NFG as a pressure regulator. We build CMOS‐inspired fluidic inverter amplifiers in Figure 4 to actively control the gain, hysteresis, and amplitude of output pressure. Unlike the pneumatic amplifiers developed by Takao et al.,^[^ ^24^ ^]^ our CMOS‐inspired amplifiers can be powered by a single positive pressure differential, providing a potential benefit of minimizing the necessary pneumatic peripherals for light‐weight soft robotic devices. Furthermore, we demonstrate multiplication of the pressure gain of the CMOS‐inspired fluidic inverters, and active control of the circuit's gain using the NFG as a pressure follower. By cascading two fluidic inverters P1N1 and P2N2 in Figure 4B, we achieve a pressure gain up to 2.6, and controlled it from 1.8 to 2.6 by changing a regulator pressure (*P*_r_) of the pressure follower. The 2.6 of pressure gain achieved in this work is certainly not the highest gain, compared to other previous works.^[^ ^20^, ^41^ ^]^ Still, the concept of gain multiplication and active control of the pressure gain is an important contribution, as it allows us to easily customize the pressure gain of fluidic amplifiers, instead of going back to a drawing board to redesign a completely new fluidic gates or circuits. The use of PFGs and NFGs as building blocks, as well as the concept of using an NFG as a pressure follower, allow us to replicate further complex CMOS electronic circuits, such as a PCO in Figure 5. The PCO demonstrates modulation of its oscillation up to 14.5 times of difference in the oscillation frequency using a single pressure input (Figure 6), and reversibly controls the crawling speed of a soft robot within a range from 0.5 to 1.0 mm s^−1^ (Figure 7). Fluidic self‐oscillators have also been demonstrated in several previous works. However, those oscillators had an oscillation frequency predetermined by circuit geometry,^[^ ^28^, ^42^, ^43^ ^]^ or a given supply pressure,^[^ ^29^, ^43^ ^]^ and maintained the oscillation until depleting the supply pressure, unlike fully controllable pneumatic oscillation demonstrated by our PCO.

The results of the proposed fluidic logic gates and their performance demonstrate their direct applicability toward advanced power fluidics for soft robots. Inspired by the power electronics, these CMOS‐inspired fluidic analog circuits can bridge the power difference between low‐power microfluidic digital logic processors and high‐power pneumatic actuators with much compact fluidic systems to empower soft robotic devices. For practical applications of such fluidic analog circuits, we list the following milestones to be achieved as future works. First of all, the PFG and the NFG need to be optimized in size to build compact and lightweight fluidic circuits as an on‐board fluidic controller for soft robots. Although the PCO in this work could successfully demonstrate the performance of controllable oscillation for the motion control of a soft earthworm robot, the circuit was not integrated with the soft robot as an on‐board controller due to its large size. Based on preliminary findings in Figure 2, it will be possible to further reduce the size of the diaphragm membrane without changing the gate's fluidic responses. We also need a design methodology for the PFG and NFG to achieve fluidic characteristics of linear response without hysteresis. As mentioned above, previous works have mainly focused on the advantages of the Schmitt trigger‐like fluidic behavior, especially its high noise resistance.^[^ ^26^ ^]^ Although the techniques developed in this work could reproduce various functionalities of analog circuits using those fluidic latching valves with hysteresis, we saw the hysteresis still problematic in the applications for the development of fluidic analog circuits. For example, the pressure follower R in Figure 4C could not regulate output pressure (*P*_o_) of the pneumatic inverter P1N1 with a gain of unity (*P*_o_ / *P*_r_ = 1), which does not match with the results of a single pressure follower R as shown in Figure 1H. Also, we saw the fluidic inverter P1N1 did not function properly at a regulator pressure (*P*_r_) less than 20 kPa in Figure 4C,4F. Figure S3, Supporting Information, shows the dynamic response of an NFG, when used as a pressure follower with respect to time. Although Figure 1H shows that the terminal amplitude of *P*_o_ can reach to that of *P*_r_ with the gain of unity, Figure S3A, Supporting Information, shows it requires up to several minutes to have *P*_o_ reached as high as *P*_r_. Also, Figure S3B, Supporting Information, shows that the current design of pressure follower can remain a residual *P*_o_, even *P*_r_ is completely removed. We speculate that Schmitt‐trigger‐like fluidic response of our fluidic gates leads to dynamic instability over the entire circuit. Therefore, we believe that the PFG and NFG linearly responsive to a control input without the fluidic hysteresis will significantly improve the performance and stability of the CMOS‐inspired fluidic circuits in the future.

In this work, we use a combination of rigid and rubber‐like materials to prototype the PFG and the NFG. The fluidic gates are assembled to be rigid overall, ensuring stable and reliable operations over repetitive testing. Although the reliable operations of the PFG and the NFG allow us to fully focus on the main purpose of this work how to design the CMOS‐inspired fluidic analog circuits, challenges still remain to fabricate the proposed PFG and NFG out of fully soft materials. As a preliminary effort shown in Figure S4, Supporting Information, we 3D‐printed a fully soft NFG using a stereolithography (Form 2, Formlabs). The 3D‐printing of the entire gate architecture out of a single material allowed us to significantly reduce the overall size by eliminating unnecessary space for manual assembly (Figure S4A, Supporting Information). Also, we observed a very interesting behavior of the v‐shaped poppet that shows the linear response with reduced fluidic hysteresis (Figure S4C, Supporting Information). Unfortunately, the NFG prototype could not be used in this work due to manufacturing imperfection caused by a limited precision of the 3D‐printer, which did not allow the valve to fully close (Figure S4B, Supporting Information). However, we believe that future advancements in additive manufacturing will eventually enable the manufacturing of 3D‐printed, fully soft fluidic gates, as well as soft pneumatic controllers. Similarly, we also see challenges and difficulties in miniaturizing the poppet‐style NFG into micro‐scale with current microfabrication techniques. However, the current progress in nano‐scale printing techniques, such as two‐photon lithography, promises that we will soon be able to fabricate intricate 3D fluidic structures in micro‐scale, which will allow us to build various CMOS‐inspired fluidic microprocessors consisting of the PFG and NFG proposed in this work.

We expect that the use of CMOS‐inspired complementary fluidic circuits as controllers for soft robots will find out unique functionalities in practical applications that conventional robotic systems couldn't achieve. As Mahon et al. pointed out, soft robots driven by pure pneumatics can be used in extreme and hazardous environments, such as offshore oil and gas environments in where electrical sparks or static electricity can cause ignition of flammable materials, leading to a catastrophic failure.^[^ ^18^ ^]^ Also, a soft fluidic actuator with fluidic circuits as a controller can be used as an MRI‐compatible manipulator for medical applications.^[^ ^44^ ^]^ Finally, compliant, lightweight, and advanced fluidic circuits will be one of the core robotic components for comprehensive, adaptive, and cohesive soft robots in the future, achieving better symbiotic integration with human or human‐centered environments.

## 4 Experimental Section

### Fabrication of Fluidic Gates

The PFG and the NFG consisted of several layers of different 3D‐printed materials, as shown in Figure S1, Supporting Information. A multi‐material printer (Connex500, Stratasys, Ltd.) was used to print the top, hole, and bottom layers with a rigid plastic (VeroClear, Stratasys, Ltd.), and the poppet and O‐ring with a rubber‐like material (TangBlackPlus/Shore A30, Stratasys, Ltd.). A multi‐material printer (Objet500 Connex3, Stratasys, Ltd.) was used to print the membrane layer with a composite material DM 9840/Shore A40 (primary material: VeroClear, secondary material: Agilus/Shore A30, Stratasys, Ltd.) for improved mechanical properties. Furthermore, a 400‐µm thick membrane core was printed with DM 9870/Shore A70 (primary material: VeroClear, secondary material: Agilus/Shore A30, Stratasys, Ltd.) inside the 800‐µm thick diaphragm membrane as a reinforcement. All layers were assembled using a commercial instant adhesive (LOCTITE 4850, Henkel AG & Company, KGaA), including silicone tubing (1.6 mm ID and 3.2 mm OD, McMaster‐Carr) on each terminal. A surface primer (LOCTITE SF 770, Henkel AG & Company, KGaA) was used to promote adhesion between the silicon tubing and 3D‐printed parts. Finally, male and female of plastic quick‐turn tube couplings (for 1.6 mm barbed tube ID, McMaster‐Carr) were used to connect the fluidic gates.

### Fabrication of SPA and Earthworm‐Like Soft Robot

This work exploited a bellow‐shaped SPA design that has been manufactured by conventional 3D printing methods in several previous works^[^ ^12^, ^45^ ^]^ for rapid evaluations of the presented fluidic circuits. Any other manufacturing methods and design of SPAs that can work with a supply pressure ranging from 15 to 50 kPa will be compatible with the fluidic circuits. The 3D‐printed SPA consisted of a front‐cap, a rear‐cap, and a bellow, as shown in Figure S5, Supporting Information. Using the Objet500 Connex3, the front‐ and rear‐cap out of the VeroClear were printed. The bellow was printed as an assembly of the skin layer and the bone layer. The skin layer was printed with the DM 9870/Shore A70, while the bone layer with the VeroClear. All three parts and silicone tubing were assembled using the LOCTITE 4850 in combination with LOCTITE SF 770. The same quick‐turn tube couplings were used to connect the SPA with a fluidic circuit.

As discussed in Figure 7A, the earthworm‐like soft crawling robot consisted of the front‐cap, rear‐cap, and bellow skin. An SLA printer (Form 2, Formlabs) was used to print all parts with a rubber‐like material (Elastic Resin, Formlabs). After thoroughly cleaned with isopropyl alcohol (IPA) for 20 min, the 3D‐printed parts were fully cured by a dedicated post‐curing machine Form Cure (Formlabs) through 20 min of UV exposure (405‐nm in wavelength) at 60 °C. Finally, all parts and silicone tubing were assembled using the LOCTITE 4850 and LOCTITE SF 770.

### Experimental Setup

The fluidic circuits presented in this work were empowered and tested with conventional off‐the‐shelf fluidic components that were compatible with a typical pressure range of SPAs for the use in real‐world applications.^[^ ^46^ ^]^ Figure S6, Supporting Information, shows the experimental set‐up. A supply air was first roughly regulated by a pressure regulator for high pressure (LP‐D‐MINI, Festo), then adjusted by a precision regulator (IR1000‐F01, SMC Co.) using a digital pressure switch (ISE30A‐01‐P, SMC Co.). A syringe pump (PHD ULTRA, Harvard Apparatus) with a 10 mL plastic syringe (14.35 mm ID, McMaster‐Carr) was used to precisely apply a control pressure to fluidic circuits. The syringe was always set to have an initial volume of 5 mL, and moved with a constant speed of 1mL min^−1^. Three pressure sensors (SSCDRRN015PDAA5, Honeywell international Inc.) and one flow sensor (AWM5104VN, Honeywell international Inc.) were used to measure changes in the circuit's fluidic characteristics at different locations. All data were collected by a voltage input module (NI 9201, National Instruments) on a DAQ chassis (cDAQ‐9178, National Instruments), and processed with a customized code written in LabView.

### Statistical Analysis

Continuous variables were expressed as mean ±1 standard deviation (SD) for four measurements (n = 4). Statistical analysis was carried out using Origin Software.

## Conflict of Interest

The authors declare no conflict of interest.

## Supporting information

Supporting Information

Supplemental Movie 1

Supplemental Movie 2

Supplemental Movie 3

Supplemental Movie 4

## Data Availability

Research data are not shared.
