# Supplementary material for: CMOS‐Inspired Complementary Fluidic Circuits for Soft Robots
Source: Adv Sci (Weinh). 2021 Aug 29;8(20):2100924. doi: 10.1002/advs.202100924 (PMC8529426; doi:10.1002/advs.202100924)
Supplement: Supplementary file 1 — Supporting Information [file ADVS-8-2100924-s001.pdf]

WILEY-VCH

© 2021 Wiley-VCH GmbH

ADVANCED SCIENCE
Open Access

Supporting Information

for *Adv. Sci.*, DOI: 10.1002/advs.202100924

CMOS-inspired Complementary Fluidic Circuits for Soft Robots

*Sukho Song, S. Joshi, and Jamie Paik**

Supporting Information

CMOS-inspired Complementary Fluidic Circuits for Soft Robots

*Sukho Song, S. Joshi, and Jamie Paik**

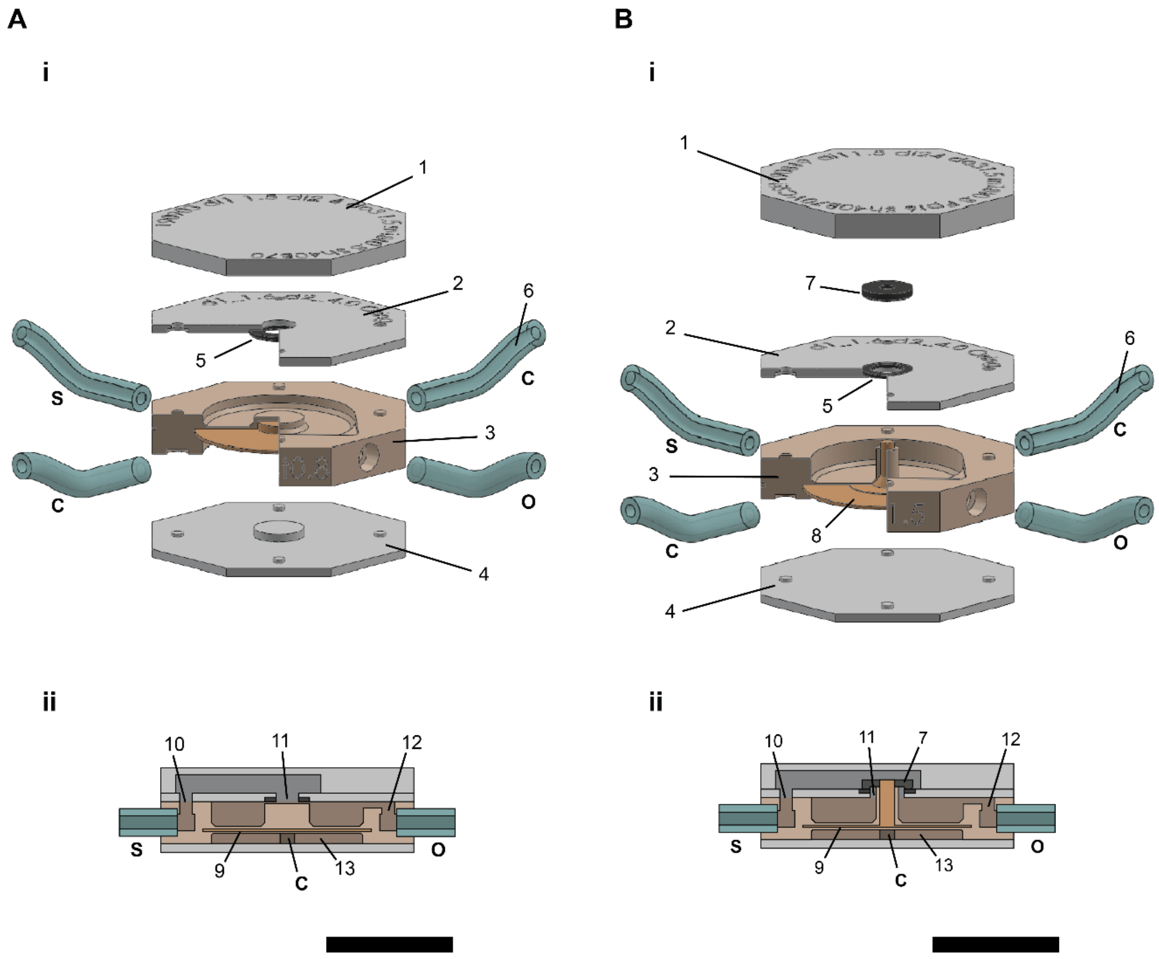

Figure S1 Parts view of a p-channel and n-channel fluidic gates. (**A-i**) A Parts view of a p-channel fluidic gate (PFG), and (**B-i**) a n-channel fluidic gate (NFG). 1: top layer (VeroClear), 2: opening layer (VeroClear), 3: membrane layer (DM 9840/Shore A40), 4: bottom layer (VeroClear), 5: o-ring (TangoBlackPlus), 6: silicone tubing, 7: poppet (TangoBlackPlus), 8: membrane bone (DM 9870/Shore A70), S: supply, O: output, C: control input. (**A-ii**) A cross-sectional view of an assembled PFG, and (**B-ii**) an assembled NFG. 9: 18 mm in diameter and 0.8 mm in thickness diaphragm membrane, 10: 1.5 mm in diameter inlet, 11: center opening with ~7.1 mm^2 of opening area (3 mm in effective diameter), 12: 1.5 mm in diameter outlet, 13: control chamber, 14: 2.6 mm in diameter poppet shaft, 15: 6 mm in diameter poppet. The scale bar corresponds to 15 mm.

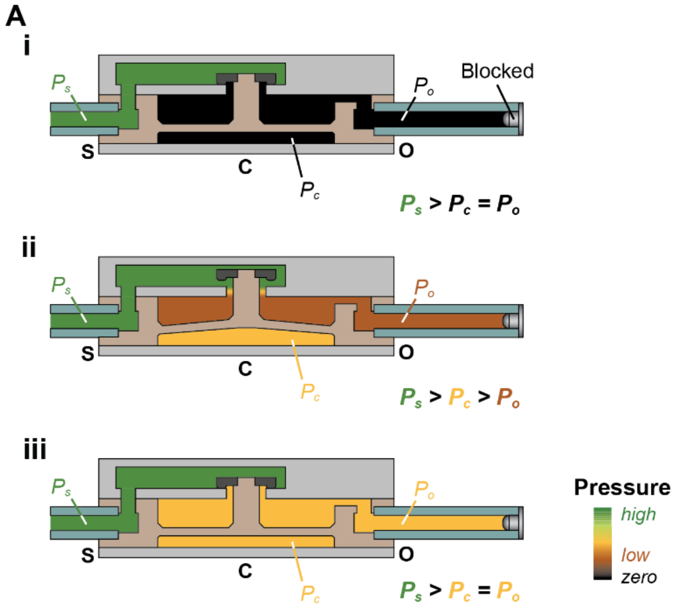

Figure S2 Schematic images of PFG and NFG in a closed circuit (output is blocked and air does not escape from the valve). (**A**) In case of an NFG, output pressure (P_o) is zero (P_o = 0) in the beginning when control pressure is zero (P_c = 0), working as a normally-OFF valve. (**i**) When P_c increases, a pressure differential across the diaphragm membrane pushes up the poppet to open the valve. (**ii**) When P_o reaches to P_c, the pressure differential disappears, leading the valve to closing the poppet. (**iii**) Therefore, the NFG can regulate its output pressure P_o, depending on the applied control pressure P_c. (**B**) In case of PFG, On the other hand, P_o and supply pressure (P_s) are the same in the beginning (P_c = 0), as the valve is normally-ON. (**i**) Since already reached to its maximum value of P_s, P_o of the PFG does not change and remains at $P_o = P_s$, regardless of P_c. (**ii** & **iii**)

Table S1 Modelled fluid resistance at inlet (r_i), center opening (r_c), and outlet (r_o) based on characterization results in inlet diameter (d_i), total pressure drop (ΔP_t), volumetric output flow rate (Q_o), and closing control pressure (P_{cc})

d_i [mm]	ΔP_t [kPa]	Q_o [L/min]	P_{cc} [kPa]	r_i [MPa·s/m^3]	r_c [MPa·s/m^3]	r_o [MPa·s/m^3]
0.8	33.2	1.6	8.8	813.5	69.6	392.5
1.2	31.2	1.8	11.0	567.8	69.6	392.5
1.5	28.9	2.0	13.3	392.5	69.6	392.5

Table S2 Oscillation frequencies (f_o) of the PCO depending on regulator pressure (P_r) and supply pressure (P_s)

P_s = 10 kPa		P_s = 20 kPa		P_s = 30 kPa		P_s = 40 kPa		P_s = 50 kPa	
P_r [kPa]	f_o [Hz]	P_r [kPa]	f_o [Hz]	P_r [kPa]	f_o [Hz]	P_r [kPa]	f_o [Hz]	P_r [kPa]	f_o [Hz]
7.26	3.68	10.02	5.79	13.24	3.73	18.68	2.79	21.65	1.57
7.82	0.92	10.35	5.01	13.73	1.25	19.86	0.88	23.18	0.66
9.19	0.15	10.88	1.55	14.93	1.05	21.35	0.74	26.42	0.61
		11.48	0.88	16.19	0.87	22.87	0.70	29.85	0.57
		12.77	0.72	17.39	0.81	24.43	0.66	33.70	0.57
		14.24	0.44	18.56	0.78	27.62	0.57	37.30	0.53
		15.54	0.42	21.20	0.63	34.29	0.57		
		17.90	0.42	25.25	0.63				

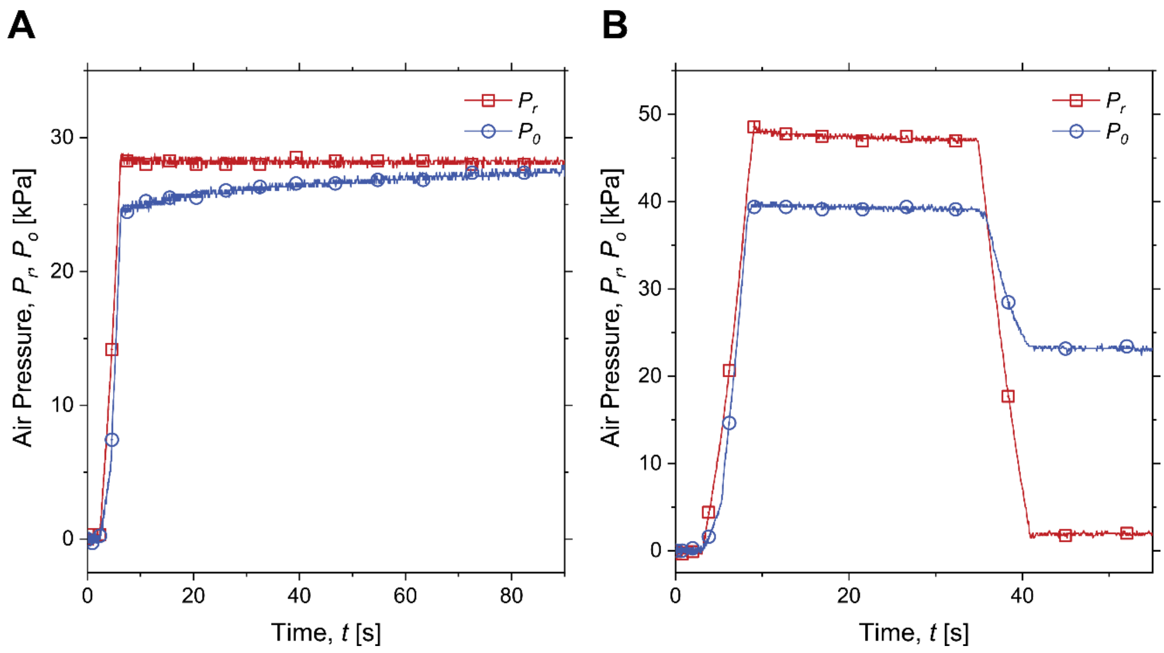

Figure S3 (**A**) Dynamic response of a NFG as a pressure follower Pressure profiles of a step input of regulator pressure (P_r), and its output pressure (P_o) of the NFG with respect to time (t). (**B**) Pressure profiles of a square wave of regulator pressure (P_r), and its output pressure (P_o) of the NFG with respect to time (t).

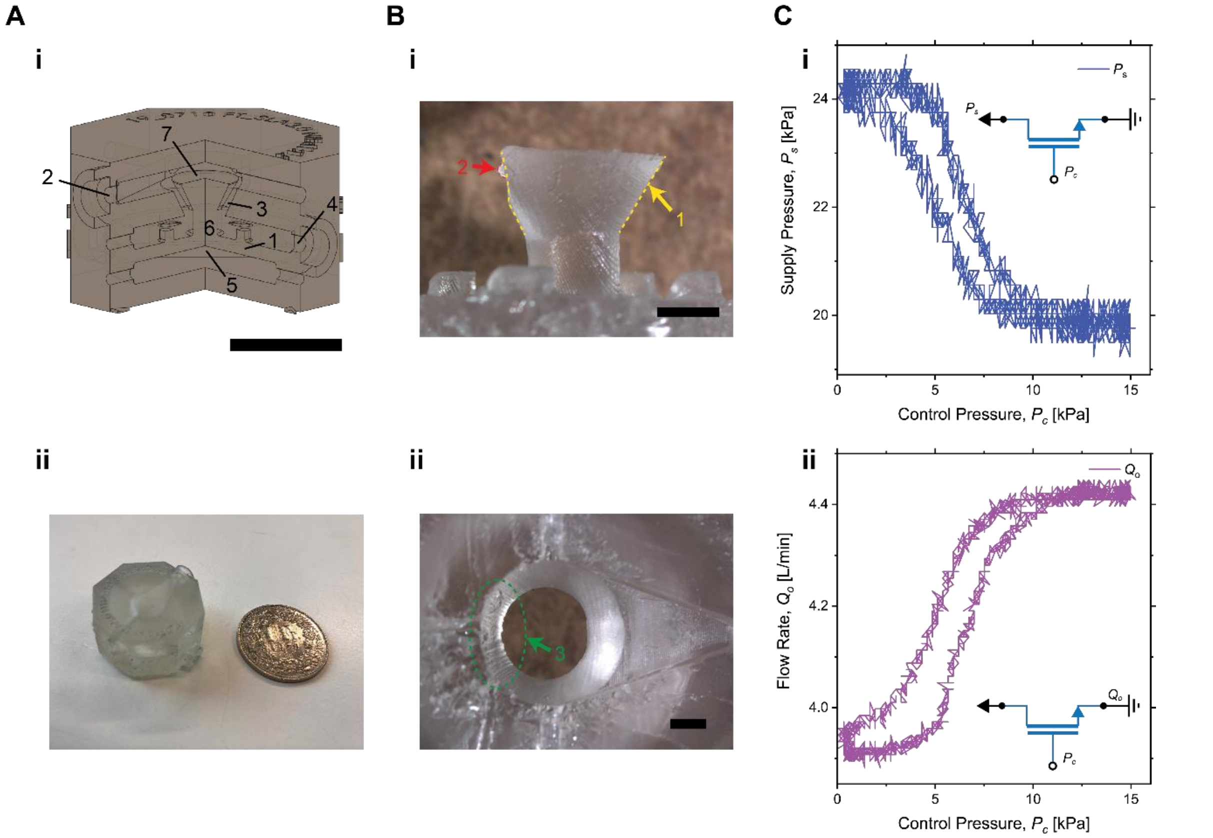

Figure S4 A prototype of fully soft NFG A cross-section of 3D assembly of the fully soft NFG (**A-i**) and a corresponding photo image of fabricated prototype (**A-ii**). 1: diaphragm membrane, 2: inlet hole, 3: center hole, 4: outlet hole, 5: control chamber, 6: poppet shaft, 7: poppet. A scale bar indicates 10 mm. Microscopic images of a side view of the v-shaped poppet (**B-i**), and a top view of the center hole (**B-ii**). 1: the v-shaped poppet is poorly defined with irregular edge, 2: the v-shaped poppet has a protrusion at the edge, 3: a part of the center hole has a rough surface that is difficult to fully close. Scale bars indicate 2 mm. Profiles of supply pressure (P_s) (**C-i**), and flow rate at output (Q_o) of the fully soft NFG (**C-ii**), depending on control pressure (P_c). The initial applied P_s is 40 kPa. Since the NFG cannot fully close a supply air, Q_o in C-ii at P_c = 0 kPa is not zero due to a leakage, causing a reduced P_s of 24 kPa at P_c = 0 kPa in C-i.

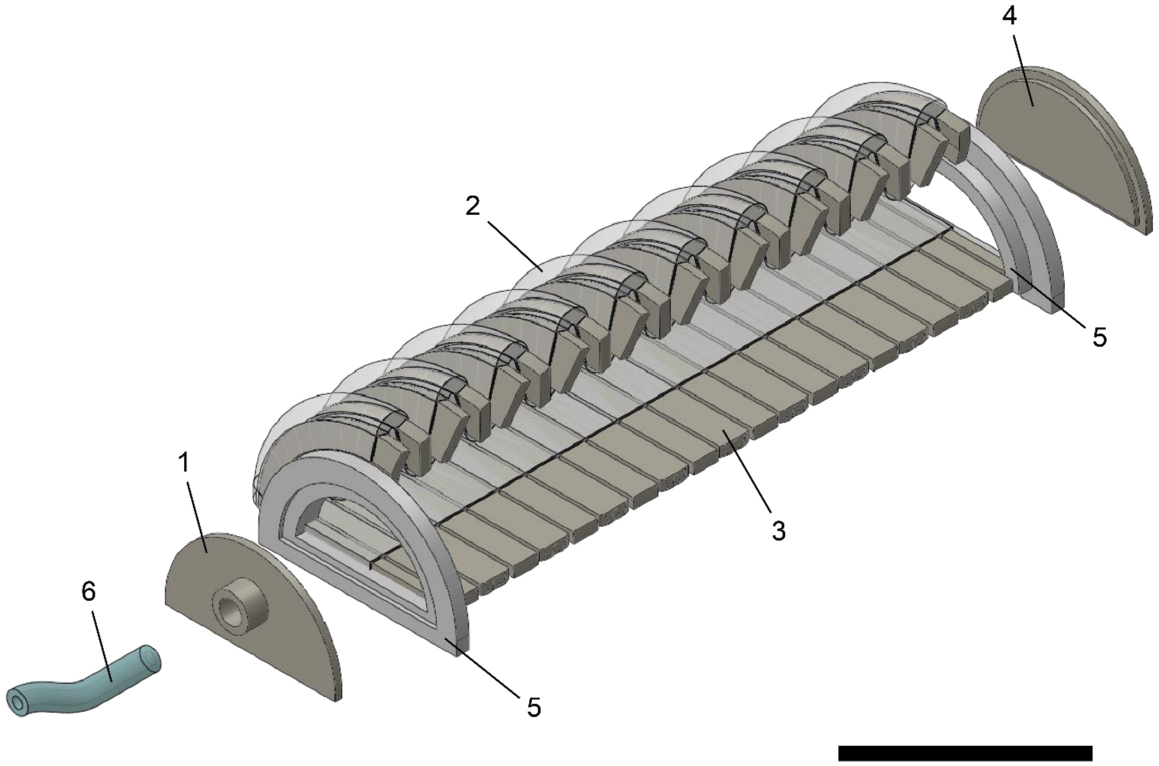

Figure S5 Parts view of a 3D-printed soft pneumatic actuator (SPA) A parts view of a SPA. 1: front-cap (VeroClear), 2: bellow skin (DM 9840/Shore A40), 3: bellow bone (VeroClear), 4: rear-cap (VeroClear), 5: cap holder (VeroClear), 6: silicone tubing. The scale bar corresponds to 30 mm.

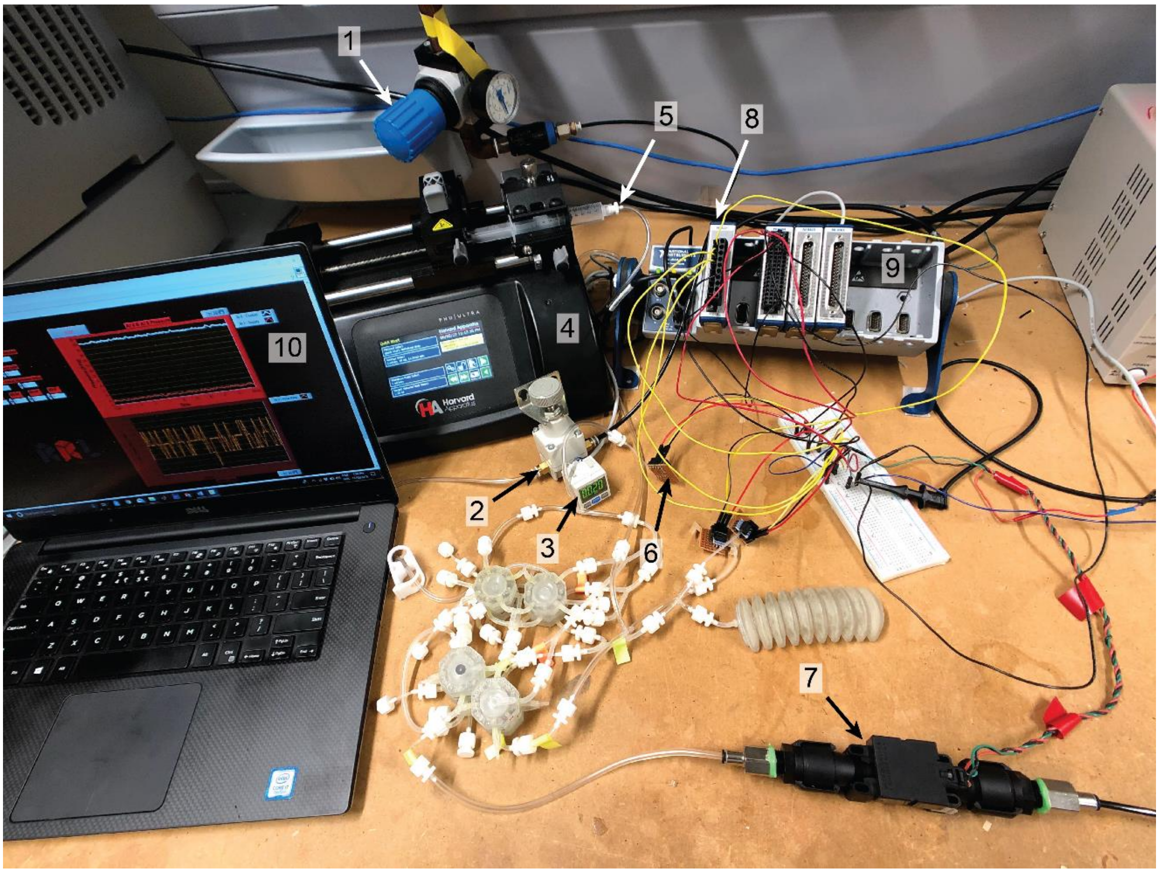

Figure S6 Experimental Set-up 1: pressure regulator for high pressure (LP-D-MINI, Festo), 2: precision pressure regulator (IR1000-F01, SMC® Co.), 3: digital pressure switch (ISE30A-01-P, SMC® Co.), 4: syringe pump (PHD ULTRA™, Harvard Apparatus), 5: plastic syringe (14.35 mm ID, McMaster-Carr®), 6: pressure sensor (SSCDRRN015PDAA5, Honeywell international Inc.), 7: flow sensor (AWM5104VN, Honeywell international Inc.), 8: voltage input module (NI 9201, National Instruments™), 9: DAQ Chassis (cDAQ-9178, National Instruments™), 10: data processing code.

Supplementary Movies

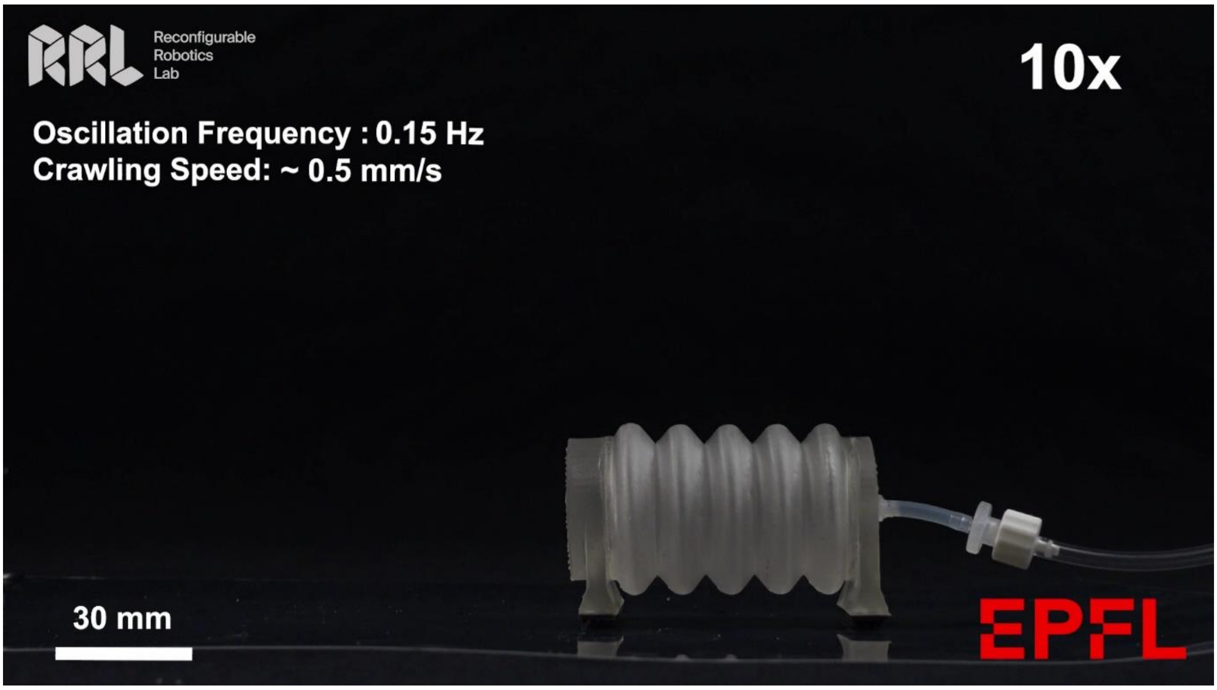

Movie S1. Controlled crawling motion of an earthworm-inspired soft robot, moving toward left side of the screen.

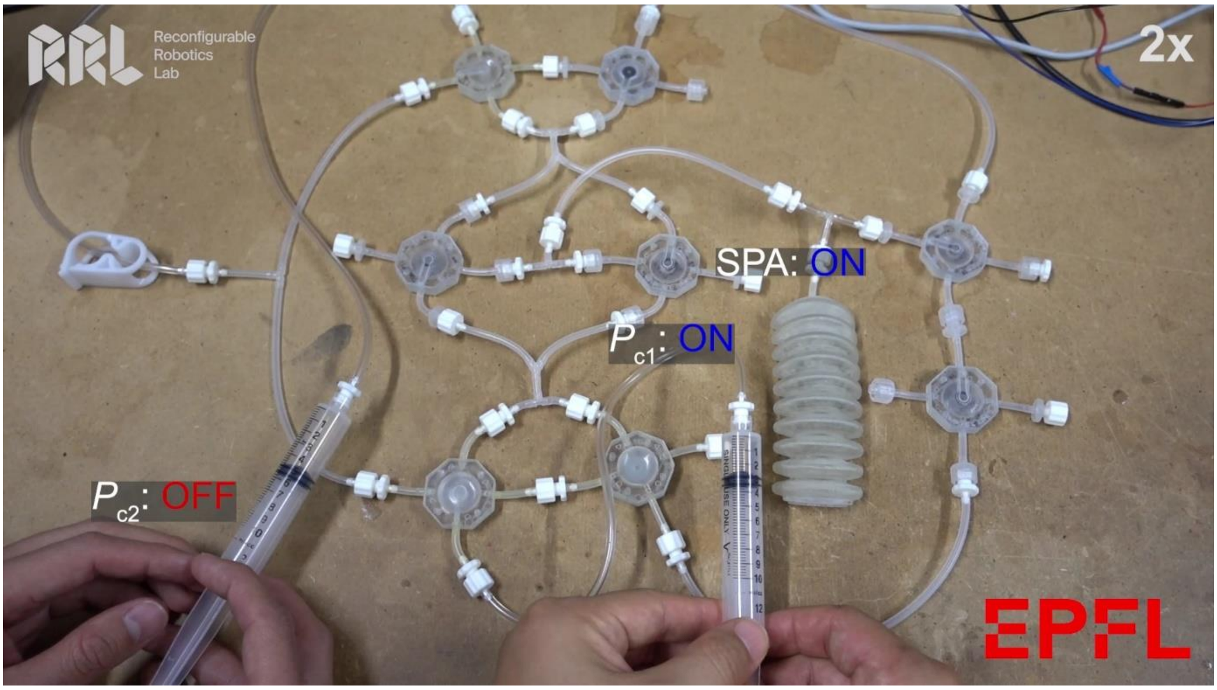

Movie S2. Actuation of a bellow-shaped soft pneumatic actuator by logic operation of the CMOS-inspired fluidic XOR logic, depending on states of two control inputs (P_{c1} and P_{c2}).

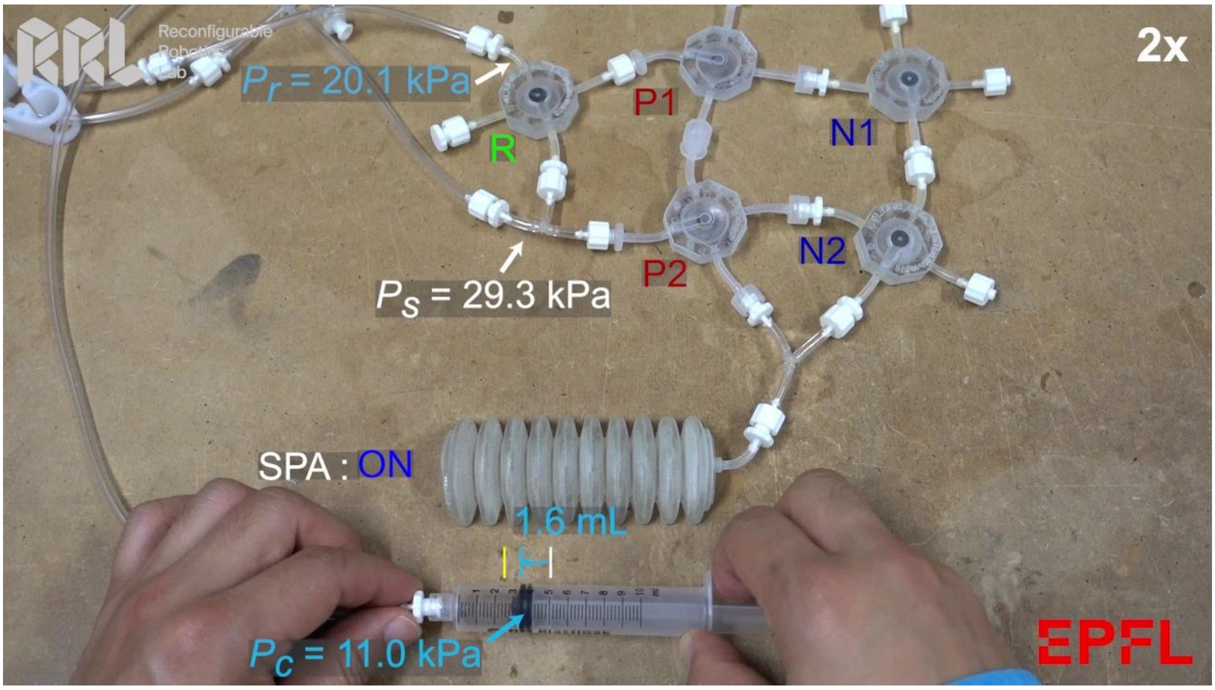

Movie S3. Active control of pressure gains between input (P_c) and output (P_o) pressure of the CMOS-inspired cascaded fluidic inverter, depending on the amount of regulator pressure (P_r).

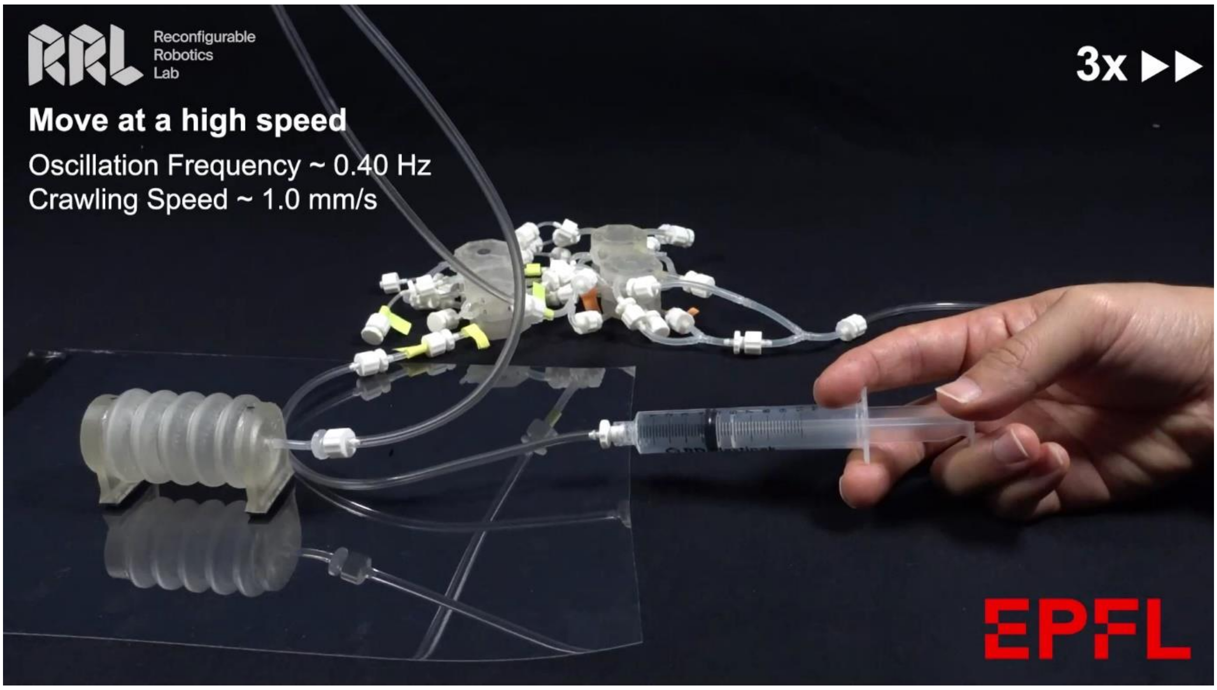

Movie S4. Demonstration of the Pressure-Controlled Oscillator (PCO) for the use in motion control of the earthworm-inspired soft robot, not only switching the robot's crawling motion on or off, but also changing its crawling speeds using a single, quasi-static input pressure.
